# The effects of probiotics supplementation on glycaemic control among adults with type 2 diabetes mellitus: a systematic review and meta-analysis of randomised clinical trials

**DOI:** 10.1186/s12967-023-04306-0

**Published:** 2023-07-06

**Authors:** Guang Li, Hao Feng, Xin-Liang Mao, Yan-Jun Deng, Xiao-Bao Wang, Qiong Zhang, Yan Guo, Su-Mei Xiao

**Affiliations:** 1 Department of Epidemiology, School of Public Health, Sun Yat-Sen University, Guangzhou, 510080 China; 2 College of Food Science and Engineering, South China University of Technology, Guangzhou, 510640 China; 3 Zhongshan Center for Disease Control and Prevention, Zhongshan, 528403 China; 4 Guangdong Provincial Key Laboratory of Food, Nutrition and Health, School of Public Health, Sun Yat-Sen University, Guangzhou, 510080 China

**Keywords:** Probiotics, Glycaemic control, Type 2 diabetes mellitus, Systematic review and meta- analysis

## Abstract

**Objective:**

This systematic review and meta-analysis study aimed to evaluate the effectiveness of probiotics supplementation on glycaemic control in patients with type 2 diabetes mellitus (T2DM) based on the data from the randomised clinical trials (RCTs).

**Methods:**

PubMed, Web of Sciences, Embase, and Cochrane Library were searched from the inception to October 2022, and RCTs about probiotics and T2DM were collected. The standardised mean difference (SMD) with 95% confidence interval (CI) was used to estimate the effects of probiotics supplementation on glycaemic control related parameters, e.g. fasting blood glucose (FBG), insulin, haemoglobin A1c (HbA1c), and homeostasis model of assessment of insulin resistance (HOMA-IR).

**Results:**

Thirty RCTs including 1,827 T2MD patients were identified. Compared with the placebo group, the probiotics supplementation group had a significant decrease in the parameters of glycaemic control, including FBG (SMD = − 0.331, 95% CI  − 0.424 to − 0.238, *P*_effect_ < 0.001), insulin (SMD = − 0.185, 95% CI  − 0.313 to − 0.056, *P*_effect_ = 0.005), HbA1c (SMD = − 0.421, 95% CI  − 0.584 to − 0.258, *P*_effect_ < 0.001), and HOMA-IR (SMD = − 0.224, 95% CI  − 0.342 to − 0.105, *P*_effect_ < 0.001). Further subgroup analyses showed that the effect was larger in the subgroups of Caucasians, high baseline body mass index (BMI ≥ 30.0 kg/m^2^), *Bifidobacterium* and food-type probiotics (*P*_subgroup_ < 0.050).

**Conclusion:**

This study supported that probiotics supplementation had favourable effects on glycaemic control in T2DM patients. It may be a promising adjuvant therapy for patients with T2DM.

**Supplementary Information:**

The online version contains supplementary material available at 10.1186/s12967-023-04306-0.

## Introduction

Type 2 diabetes mellitus (T2DM), an endocrine and metabolic disease, is influenced by host physiology and environmental factors [1]. More than 500 million people are living with diabetes globally, and this number is expected to increase to 783 million by 2045 [2]. T2DM is a common disease that accounts for approximately 90% of all cases of diabetes [3], and it may cause reduced life expectancy and life-threatening and costly complications [4]. There is no radical cure for T2DM [5, 6], and its treatment relies on the long-term use of anti-diabetic drugs [7, 8]. Therefore, it is crucial to explore new methods that may effectively delay or even reverse the progression of T2DM.

Recent studies have shown that the gut microbiota plays a key role in the maintenance of host homeostasis and pathogenesis of T2DM [9, 10]. Probiotics are microbial dietary supplements that alter the gut microbiota. Some randomised controlled trials (RCTs) have investigated the effects of probiotic interventions on glycaemic control in T2DM patients. However, evidence from clinical trials on the effects of probiotic supplementation on glycaemic control remains inconsistent. Asemi et al. [11] conducted a randomised double-blind placebo-controlled clinical trial involving 54 T2DM patients, which revealed that multi-species probiotic (mixture of *Lactobacillus* and *Bifidobacterium*) supplementation prevented an increase in the fasting blood glucose (FBG) level from baseline in these patients. Meanwhile, Razmpoosh et al. [12] randomly assigned 60 T2DM patients into two groups to take either a probiotic (mixture of *Lactobacillus* and *Bifidobacterium*) or a placebo intervention, and the results showed no significant differences in insulin or insulin resistance levels between the two groups. In 2016, Li et al. performed a systematic review and meta-analysis of 12 RCTs with 714 individuals and reported that probiotic supplementation could alleviate FBG, but no significant differences were observed in the haemoglobin A1c (HbA1c) level or homeostatic model assessment of insulin resistance (HOMA-IR) score between the probiotic and control groups of T2DM patients [13]. In 2020, Tao et al. systematically summarised 15 RCTs with 902 individuals, and the results of the meta-analysis indicated that probiotic supplementation reduced HbA1c, FBG and insulin resistance levels in T2DM patients [14]. However, some related RCTs (*n* = 11, including 630 patients) were not included in their study. Since then, more RCTs (*n* = 6) of the effects of probiotic supplementation on glycaemic control, including a total of 511 T2DM patients, have been reported [15, 16]. Controversy still exists regarding the effects of probiotics on glycaemic control in T2DM patients. Variations in participant (e.g. race) and intervention characteristics (e.g. dose, probiotic genus, and duration) in different studies may have given rise to the contradictory results. No study has detected differences in the effects of probiotic supplementation on glycaemic control according to the participant and intervention characteristics.

In this systematic review and meta-analysis, we aimed to evaluate the effects of a probiotic intervention on glycaemic control in T2DM patients and to evaluate the variations in these effects due to participant characteristics, e.g. race and baseline body mass index (BMI), and intervention characteristics, e.g. the probiotic dose, the duration of the intervention, the probiotic genus, and the type of vehicle used to deliver the probiotics.

## Methods

This study followed the Preferred Reporting Items for Systematic Reviews and Meta-Analyses statement [17] (Additional file 1: Table S1). The protocol for this study has been registered at the International Prospective Register of Systematic Reviews (registration number: CRD42022370226).

### Search strategy

Two reviewers (Guang Li and Yan-Jun Deng) independently searched PubMed, Web of Science, Embase, and Cochrane Library databases from their inception until October 2022 using various probiotic-related words and Medical Subject Heading terms in combination with ‘T2DM’ (Additional file 2: Table S2). No language or other restrictions were applied during the search, and all relevant studies were found to be published in English. A manual search was also performed to identify relevant studies from the references of the included studies.

### Inclusion and exclusion criteria

Studies were included in the analysis if: (1) the participants were T2DM patients aged ≥ 18 years; (2) the study design was an RCT; (3) the intervention was the intake of probiotics from supplements and/or food; (4) the control group received a placebo intervention; and (5) the main outcomes included the glycaemic profile, e.g. FBG, insulin, and HbA1c levels and the HOMA-IR score. Studies were excluded from the analysis if: (1) the participants had other types of diabetes, e.g. gestational diabetes or type 1 diabetes or (2) the participants were concurrently receiving other interventions, e.g. synbiotics, herbs, prebiotics, or micro- nutrients.

### Data extraction and quality assessment

Two researchers (Guang Li and Yan-Jun Deng) independently performed the literature search and data extraction, and disagreements were resolved by a third senior researcher (Su-Mei Xiao). Basic information (e.g. first author, year, and country of the study and the age, sex, and BMI of the participants), the study design, intervention information (probiotic genus and dose and duration of the intervention), and outcomes were extracted from the included studies. Two reviewers (Xiao-Bao Wang and Qiong Zhang) evaluated the quality of the included studies using the Cochrane risk-of-bias assessment tool. The risk of bias in the included studies was classified as low, unclear, or high.

### Data synthesis and statistical analysis

The change in glycaemic control parameters was the primary outcome in this study. It was calculated as the final measurement value minus the baseline measurement value in each group. The mean and standard deviation (SD) of the change in glycaemic control parameters for the control group and the intervention group were extracted, respectively. If the study provided the standard error (SE) of mean change, the SE was converted to SD based on the sample size. For studies that did not directly report SD of mean change, the SDs of the baseline and final measurement values and the correlation coefficient (*Corr*) were used to calculated SD_Effect,change_ (SD_E,change_) according to the following formula [18]:

$${\mathrm{SD}}_{\mathrm{E},\mathrm{change}}=\sqrt{{SD}_{E,baseline}^{2}+{SD}_{E,final}^{2}-(2 * Corr * {SD}_{E,baseline} * {SD}_{E, final})}$$

*Corr* is the correlation coefficient between the baseline and final measurement values. For the pretest–posttest design, presumably the correlation is at least 0.5. This was the *Corr* estimate value being used to impute the missing SDs of mean change in this study [18, 19]. If the study presented data in medians and quartiles, the mean and SD values were estimated [20, 21]. If the intervention included multiple time points, the longest intervention time was included in the analysis.

The standardised mean difference (SMD) with the 95% confidence interval (CI) was used to assess the effects of probiotic interventions on glycaemic control in T2DM patients. The boundary values of the SMD were set at 0.2, 0.5, and 0.8, corresponding to small, medium, and large effects, respectively [22]. Heterogeneity was assessed using Cochrane’s Q statistic (chi-square). The inverse variance (*I*^2^) was used to assess the size of the heterogeneity. A fixed-effects model was used for the meta-analysis when *I*^2^ ≤ 50%, and a random-effects model was used when *I*^2^ ≥ 50%. Subgroup analysis was used to explore the possible sources of heterogeneity. Subgroup analyses were performed for race (Asian vs. Caucasian), probiotic dose (≤ 1 × 10^10^ colony-forming units (CFU)/day vs. > 1 × 10^10^ CFU/day), the duration of the intervention (≤ 8 weeks vs. > 8 weeks), probiotic genus (*Lactobacillus*, *Bifidobacterium*, or *Lactobacillus* and *Bifidobacterium*), type of vehicle used to deliver the probiotics (food vs. non-food (powder/capsule/tablet), and baseline BMI (< 30 kg/m^2^ vs. ≥ 30 kg/m^2^). The leave-one-out approach was used in the sensitivity analysis. Funnel plots and Egger’s test were used to appraise the possible publication bias in this study.

## Results

### Study characteristics

**Fig. 1:**
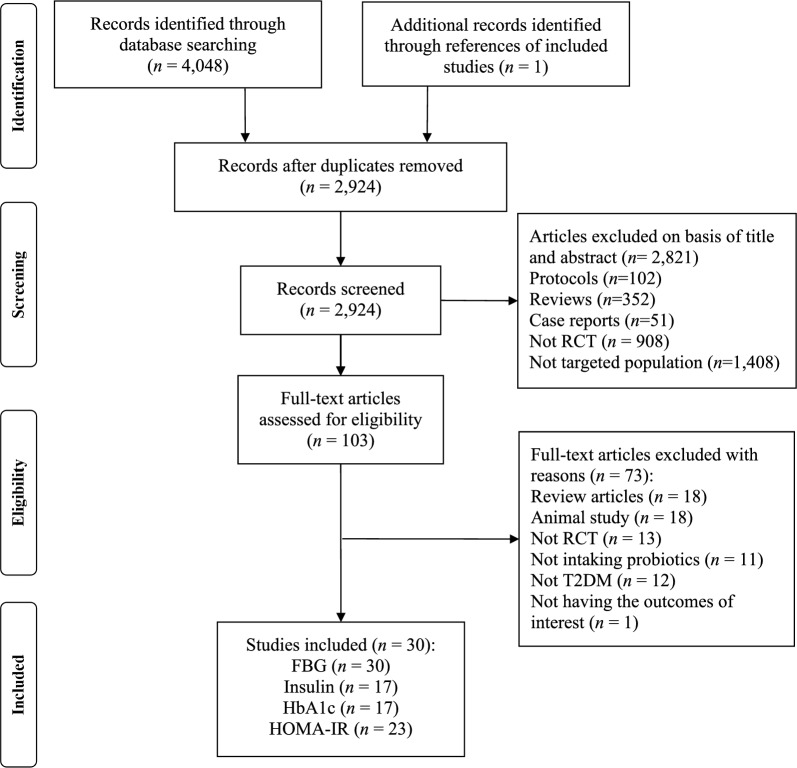
PRISMA flowchart for search strategy and study selection process. RCT, randomised controlled trial; T2DM, type 2 diabetes mellitus; PRISMA, preferred reporting items for systematic reviews and meta-analyses; FBG, fasting blood glucose; HbA1c, haemoglobin A1c; HOMA-IR, homeostasis model of assessment of insulin resistance

The database search yielded 4,048 records, and one additional record (a conference paper [23]) was obtained from the manual search of the references of the included RCTs. A total of 1,125 records were then excluded due to duplication, leaving 2,924 articles for screening. After the screening based on the titles and abstracts, 2,821 articles were further excluded (e.g. reviews, protocols, animal studies, etc.). The full texts of the remaining 103 potentially relevant studies were assessed according to the inclusion and exclusion criteria. Finally, thirty RCTs were included in this systematic review and meta-analysis (Fig. 1).

For the included 30 RCTs, all of them reported FBG, 17 RCTs reported HOMA-IR, 17 RCTs reported insulin, and 23 RCTs reported HbA1C (Fig. 1). Table 1 shows the basic information for the included 30 studies. Nine studies were conducted in Asian patients (three in China [15, 16, 24] and one each in India [25], Indonesia [26], Thailand [27], Japan [28], Malaysia [29], and Korea [30]), 19 studies were conducted in Caucasian patients (12 in Iran [11, 12, 31–40] and one each in Ukraine [41], Turkey [23], Sweden [42], Saudi Arabia40 [43], Egypt [44], Denmark [45], and Australia [46]) and two studies were conducted in other races (two in Brazil [47, 48]). In the 30 RCTs, there were a total of 1,827 subjects, with 922 in the probiotic group and 905 in the control group. The dose of probiotics used in the 30 studies ranged from 2 × 10^7^ to 1 × 10^12^ CFU/day, the duration of the probiotic interventions ranged from 4 to 36 weeks, and the baseline BMI ranged from 23.1 to 35.9 kg/m^2^. The probiotics were consumed as food (*n* = 13) or non-food (powder/capsule/tablet; *n* = 15) forms, and the probiotic genera were mainly *Lactobacillus* (*n* = 11), *Bifidobacterium* (*n* = 2), and *Lactobacillus* and *Bifidobacterium* (*n* = 14; Table 1).

**Table 1:** Characteristics of the included studies (n = 30)

First author, year	Country	Sample size		Age, years (mean ± SD)		BMI, kg/m^2^ (mean ± SD)		Study design	Type of vehicles for probiotics	Probiotics (genus and daily dose)	Duration (weeks)
		P	C	P	C	P	C				
Toejing, 2021 [27]	Thailand	18	18	63.5 ± 5.9	61.8 ± 7.7	23.22 ± 2.72	23.05 ± 2.60	DB, PC	Powder	*L. paracasei HII01* (5 × 10^10^ CFU/day)	12
Zhang, 2020 [15]	China	102	103	52.6 ± 10.5	53.6 ± 11.3	25.60 ± 2.96	26.20 ± 3.43	DB, PC	Powder	Probiotics mixture of (5 × 10^10^ CFU/day, *Bifidobacterium longum BL88-Onlly, Bifido -bacterium breve BB8, Lactococcus gasseri LG23, Lactobacillus rhamnosus LR22, Lactobacillus salivarius LS86, Lactobacillus crispatus LCR15, Lactobacillus plantarum LP-Onlly, Lactobacillus fermentum LF33, Lactobacillus casei LC18*)	13
Palacios, 2020 [46]	Australia	30	30	61.4 ± 8.9	56.1 ± 12.3	35.50 ± 6.20	36.30 ± 7.50	DB, PC	Capsule	Probiotics mixture of *Lactobacillus plantarum Lp-115 1* (2 × 10^10^ CFU/day)*, Lacto -bacillus bulgaricus Lb-64* (6 × 10^9^ CFU/day), *Lactobacillus gasseri Lg-36* (3.6 × 10^10^ CFU/day), *Bifidobacterium breve Bb-03* (1.5 × 10^10^ CFU/day)*, Bifidobacterium animalis sbsp. lactis Bi-07* (1.6 × 10^10^ CFU/day), *Bifidobacterium bifidum Bb-06* (1.4 × 10^10^ CFU/day), *Streptococcus thermophilus St-21* (9 × 10^8^ CFU/day), *Saccharomyces boulardii DBVPG 6763* (9 × 10^7^ CFU /day)	12
Lsmail, 2020 [44]	Egypt	50	50	48.3 ± 12.9	46.4 ± 13.2	31.10 ± 5.30	30.20 ± 6.00	PC	Yogurt	*Bifidobacterium animalis dn-173 010* (NR)	16
Tipici, 2020 [23]	Turkey	17	17	NR	NR	35.51 ± 7.33	33.65 ± 6.17	PC	NR	*Lactobacillus GG* (1 × 10^10^ CFU/day)	8
Jiang, 2020 [16]	China	42	34	56.0 ± 8.5	56.1 ± 8.2	27.51 ± 3.22	26.44 ± 2.78	DB, PC	Capsule	Probiotics mixture of (*Bifidobacterium bifidum* (1.2 × 10^9^ CFU/day), *Lactobacillus acidophilus* (4.2 × 10^9^ CFU/day), *Streptococcus thermophilus* (4.3 × 10^9^ CFU/day))	12
Sabico, 2019 [43]	Saudi Arabia	31	30	48.0 ± 8.3	46.6 ± 5.9	30.10 ± 5.00	29.40 ± 5.20	DB, PC	Powder	Probiotics mixture of (4 × 10^9^ CFU/day, *Bifidobacterium bifidum W23, Bifidobacterium lactis W52, Lactobacillus acidophilus W37, Lactobacillus brevis W63, Lactobacillus casei W56, Lactobacillus salivarius W24, Lactococcus lactis W19* and *Lactobacillus lactis W58*)	24
Razmpoosh, 2019 [12]	Iran	30	30	58.6 ± 6.5	61.3 ± 5.2	27.70 ± 4.20	27.20 ± 4.20	DB, PC	Capsule	Probiotics mixture of (*Lactobacillus acidophilus* (4 × 10^9^ CFU/day) *Lactobacillus casei* (1.4 × 10^10^ CFU/day), *Lactobacillus rhamnosus* (3 × 10^9^ CFU/day), *Lactobacillus bulgaricus* (4 × 10^8^ CFU/day), *Bifidobacterium breve* (6 × 10^10^ CFU/day), *Bifidobacterium longum* (1.4 × 10^10^ CFU/day), *Streptococcus thermophilus* (3 × 10^9^ CFU/day))	6
Khalili, 2019 [33]	Iran	20	20	44.0 ± 8.1	45.0 ± 5.4	29.50 ± 3.34	31.94 ± 5.76	DB, PC	Capsule	*cfu L. casei* (1 × 10^8^ CFU/day)	8
Madempudi, 2019 [25]	India	40	39	54.1	50.6	NR	NR	DB, PC	Capsule	Probiotics mixture of (6 × 10^10^ CFU/day, *L. salivarius UBLS22, L. casei UBLC42, L. plantarum UBLP40, L. acidophilus UBLA34, B. breve UBBr01,* and *B. coagulans Unique IS2*)	12
Raygan, 2018 [39]	Iran	30	30	60.7 ± 9.4	61.8 ± 9.8	30.30 ± 5.20	29.30 ± 4.10	DB, PC	Capsule	Probiotics mixture of (*Bifidobacterium bifidum* (2 × 10^9^ CFU/day), *Lactobacillus casei* (2 × 10^9^ CFU/day), *Lactobacillus acidophilus* (2 × 10^9^ CFU/day))	12
Hsieh, 2018 [24]	China	22	22	52.3 ± 10.2	55.8 ± 8.6	28.04 ± 4.29	27.53 ± 3.15	DB, PC	Capsule	*Lactobacillus reuteri ADR-1* (4 × 10^9^ CFU/day)	36
Kobyliak, 2018 [41]	Ukraine	31	22	52.2 ± 1.7	57.18 ± 2.06	34.70 ± 1.29	35.65 ± 1.57	DB, PC	NR	Probiotics mixture of (*Lactococcus* (6 × 10^11^ CFU/day), *Bifidobacterium* (1 × 10^11^ CFU/day), *Propionibacterium* (3 × 10^11^ CFU/day), *Acetobacter* (1 × 10^7^ CFU/day))	8
Sato, 2017 [28]	Japan	34	34	64.0 ± 9.2	65.0 ± 8.3	24.20 ± 2.60	24.60 ± 2.60	DB, PC	Fermented milk	*Lactobacillus casei strain Shirota* (4 × 10^10^ CFU/day)	16
Mobini, 2017 [42]	Sweden	14	15	64.0 ± 6.0	65.0 ± 5.0	32.30 ± 3.40	30.70 ± 4.00	DB, PC	Tablet	*Lactobacillus reuteri DSM 17938* (1 × 10^10^ CFU/day)	12
Firouzi, 2017 [29]	Malaysia	48	53	52.9 ± 9.2	54.2 ± 8.3	29.20 ± 5.60	29.30 ± 5.30	DB, PC	Powder	Probiotics mixture of (6 × 10^10^ CFU/day, *Lactobacillus acidophilus, Lactobacillus casei, Lactobacillus lactis, Bifidobacterium bifidum, Bifidobacterium longum, Bifidobacterium infantis*)	12
Feizollahzadeh, 2017 [32]	Iran	20	20	56.9 ± 8.1	53.6 ± 7.2	26.68 ± 3.18	26.58 ± 3.24	DB, PC	Soy milk	*Lactobacillus planetarum A7* (2 × 10^7^ CFU/day)	8
Tonucci, 2017 [47]	Brazil	23	22	51.8 ± 6.6	51.0 ± 7.2	27.49 ± 3.97	27.94 ± 4.15	DB, PC	Fermented milk	Probiotics mixture of (*Lactobacillus acidophilus La-5* (1 × 10^9^ CFU/day), *Bifidobacterium animalis subsp. lactis BB-12* (1 × 10^9^ CFU/day))	6
Bayat, 2016 [31]	Iran	20	20	54.1 ± 9.5	47.0 ± 9.3	28.77 ± 4.59	29.75 ± 4.66	PC	Yogurt	NR	8
Ostadrahimi, 2015 [48]	Brazil	30	30	NR	NR	28.89 ± 4.77	27.47 ± 3.55	DB, PC	Fermented milk	*Bifidobacterium animalis HN019 2.7* (2 × 10^10^ CFU/day)	8
Hove, 2015 [45]	Denmark	23	18	58.5 ± 7.7	60.6 ± 5.2	29.20 ± 3.80	27.70 ± 3.30	DB, PC	Fermented milk	*Lactobacillus helveticus Cardi04* (NR)	12
Tajadadi-Ebrahimi, 2014 [36]	Iran	27	27	52.0 ± 7.2	53.4 ± 7.5	29.80 ± 5.70	30.50 ± 4.10	DB, PC	Bread	*Lactobacillus Sporogenes* (3 × 10^8^ CFU/day)	8
Shakeri, 2014 [35]	Iran	26	26	52.3 ± 8.2	53.1 ± 7.5	29.50 ± 5.70	30.60 ± 4.10	DB, PC	Bread	*L. Sporogenes* (1.2 × 10^10^ CFU/day)	8
Mohamadshahi, 2014 [34]	Iran	22	22	53.0 ± 5.9	49.0 ± 7.1	28.36 ± 4.14	29.22 ± 3.20	DB, PC	Yogurt	Probiotics mixture of (*Lactobacillus acidophilus* (1.11 × 10^9^ CFU/day), *Bifidobacterium lactic* (1.11 × 10^9^ CFU / day))	8
Jung, 2014 [30]	Korea	21	20	63.3 ± 9.2	60.2 ± 8.5	25.90 ± 4.12	25.60 ± 3.13	DB, PC	Milk	probiotics mixture of (*Lactobacillus acidophilus* (3 × 10^10^ CFU/day), *Lactobacillus casei* (1.8 × 10^10^ CFU/day), *Bifidobacterium lactis* (9.6 × 10^10^ CFU/day), *Streptococcus thermophilus* (NR))	8
Judiono, 2014 [26]	Indonesian	36	36	NR	NR	NR	NR	PC	Milk	Probiotics mixture of (*lactic acid bacter*ias (2 × 10^9^ CFU/day), and other 34 beneficial heal -thy probiotic bacterias (NR))	4
Mazloom, 2013 [38]	Iran	16	18	55.4 ± 8.0	51.8 ± 10.2	27.97 ± 3.81	27.24 ± 2.73	SB, PC	Capsule	Probiotics mixture of (*Lactobacillus acidophilus* (NR), *Lactobacillus bulgaricus* (NR), *Lactobacillus bififi dum* (NR), *Lactobacillus casei* (NR))	6
Asemi, 2013 [11]	Iran	27	27	50.5 ± 9.8	52.6 ± 7.1	31.61 ± 6.36	30.17 ± 4.23	DB, PC	Capsule	probiotics mixture of (*L. acidophilus* (2 × 10^9^ CFU/day), *L. casei* (7 × 10^9^ CFU/day), *L. rha -mnosus* (1.5 × 10^9^ CFU/day), *L. bulgaricus* (2 × 10^8^ CFU/day), *Bifidobacterium breve* (2 × 10^10^ CFU/day), *B. longum* (7 × 10^9^ CFU/day), *Streptococcus thermophilus* (1.5 × 10^9^ CFU/day))	8
Hosseinzadeh, 2013 [40]	Iran	42	42	46.8 ± 6.2	45.7 ± 6.1	30.00 ± 4.40	29.90 ± 4.70	DB, PC	Tablet	NR	12
Ejtahed, 2012 [37]	Iran	30	30	50.9 ± 7.7	51.0 ± 7.3	28.95 ± 3.65	29.14 ± 4.30	DB, PC	Yogurt	Probiotics mixture of (*Lactobacillus acidophilus La5* (2.2 × 10^9^ CFU), *Bifidobacterium lactis Bb12* (1.8 × 10^9^ CFU), *Lactobacillus bulgaricus* (NR), *Streptococcus thermophilus* (NR))	6

P, probiotic group; C, control group; DB, double-blinded; PC, placebo-controlled; SB, single blinded; NR, not reported; CFU, colony-forming units

### Risk of bias assessment of the included RCTs

The Cochrane risk-of-bias assessment tool was used to assess the bias of the 30 included studies. Approximately half of the studies (53%) were randomised, but 14 studies did not clearly report the randomisation process. The methods of allocation concealment were described in 43% of the included RCTs, and the majority of the studies (87%) described the blinding method. Approximately 40% of the studies provided information about the blinding outcome assessment. Most of the included studies had a low risk of attrition bias (73%), a low risk of reporting bias (93%), and a low risk of other types of bias (70%). Overall, four of the studies were classified as high quality (all terms were assessed as low risk), 19 studies were classified as moderate quality (no term was assessed as a high risk and one or more terms were assessed as unclear risks), and seven studies were classified as low quality (one or more terms were assessed as a high risk). The general and individual risks of bias are shown in Additional file 3: Fig. S1.

### Effects of probiotic supplementation on glycaemic control

#### Effects on FBG

**Fig. 2:**
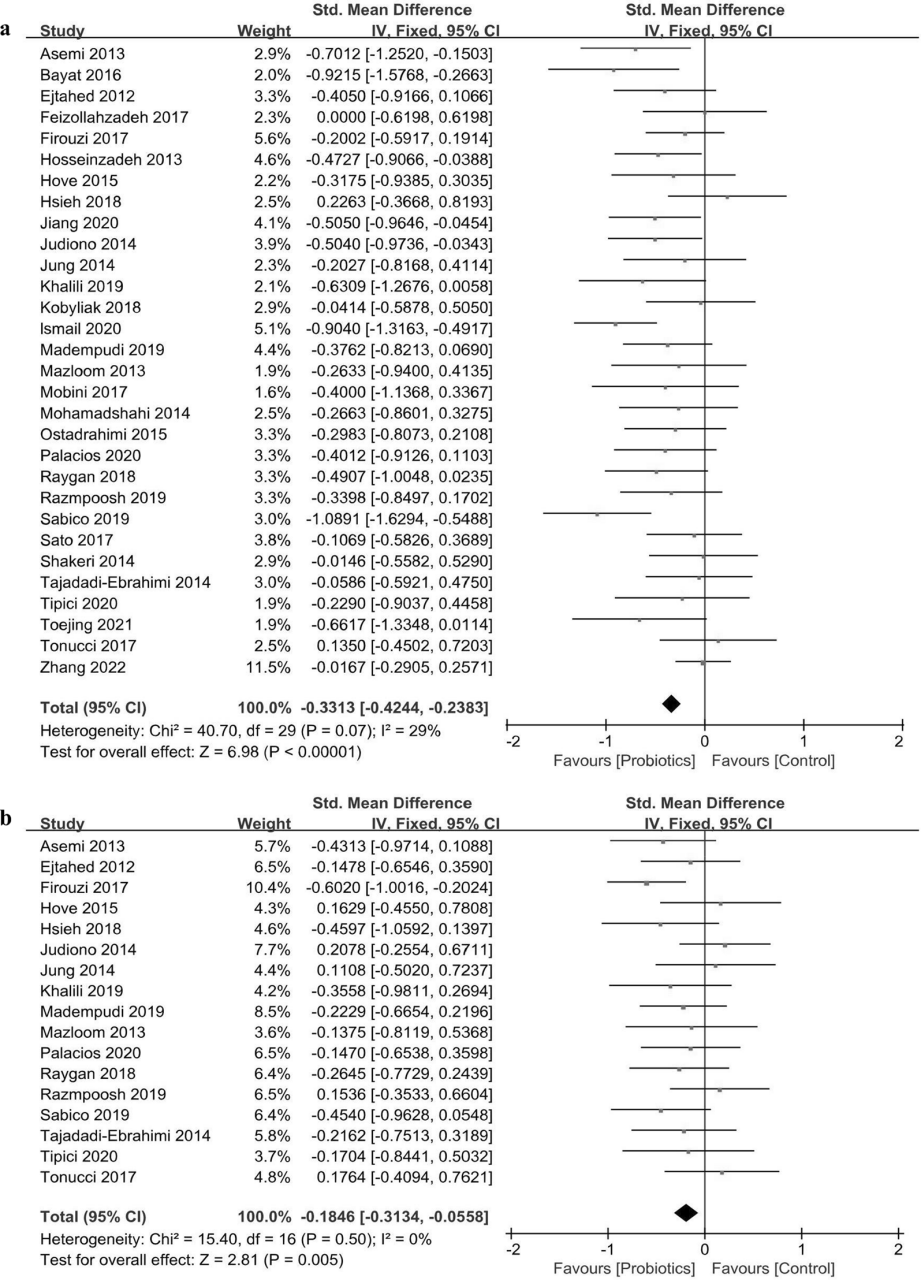

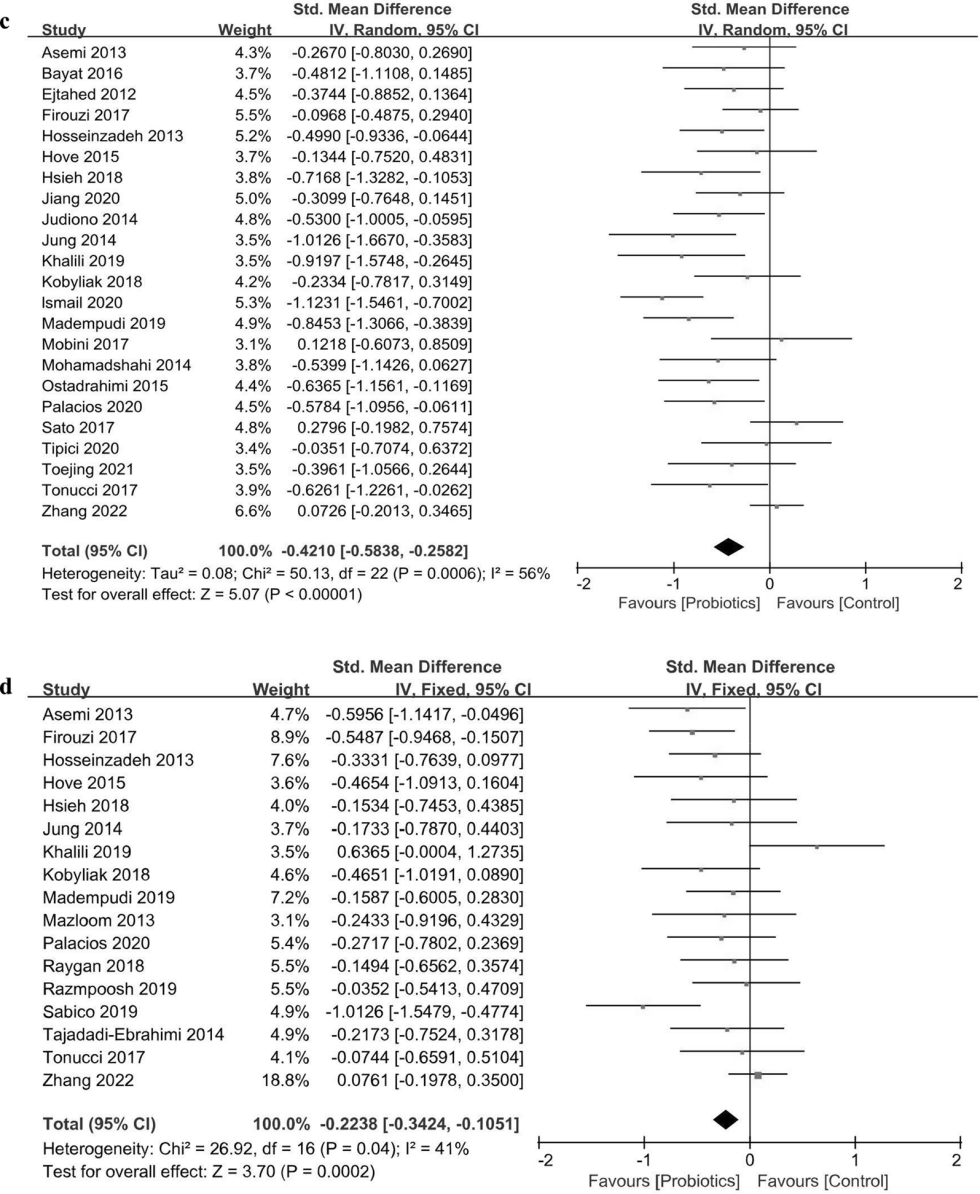
Forest plots of the effects of probiotics on **a** FBG, **b** Insulin, **c** HBA1c and **d** HOMR-IR. FBG, Fating blood glucose; HbA1c, Haemoglobin A1c; HOMA-IR, Homeostsis model of assessment of insulin resistance

Thirty studies including a total of 1,827 T2DM patients were used to evaluate the effects of probiotic supplementation on FBG level. The pooled effects of probiotic supplementation indicated a significant decrease in FBG level in the probiotic group (SMD = − 0.331, 95% CI  − 0.424 to − 0.238, *P*_effect_ < 0.001), and the heterogeneity was low (*I*^2^ = 29%, *P*_heterogeneity_ = 0.070; Fig. 2a). Leave-one-out sensitivity analysis confirmed that the pooled effects of probiotic supplementation on FBG level were stable and reliable (Additional file 4: Fig. S2a).

Subgroup analyses for FBG were performed according to race, probiotic intervention dose, probiotics genus, type of vehicle used to deliver the probiotics, and baseline BMI. As shown in Table 2, the significant subgroup differences (*P*_subgroup_ < 0.050) were observed for races (Asian vs. Caucasian), genus of probiotics (*Lactobacillus* vs. *Bifidobacterium* vs. *Lactobacillus* and *Bifidobacterium*), and baseline BMI (< 30 kg/m^2^ vs. ≥ 30 kg/m^2^). A stronger beneficial effect of the probiotic intervention was observed on FBG level in the Caucasian subgroup (SMD = − 0.448, 95% CI  − 0.575 to − 0.322, *P*_effect_ < 0.001, *P*_subgroup_ = 0.020), in the *Bifidobacterium* subgroup (SMD = − 0.626, 95% CI  − 1.221 to − 0.030, *P*_effect_ = 0.039, *P*_subgroup_ = 0.040), and in the high-baseline-BMI (≥ 30 kg/m^2^) subgroup (SMD = -0.490, 95% CI  − 0.644 to − 0.336, *P*_effect_ < 0.001, *P*_subgroup_ = 0.007). No differences were observed between the subgroups of probiotic dose, intervention duration, or type of vehicle used to deliver the probiotics (Table 2, *P*_subgroup_ > 0.050).

**Table 2:** Subgroup analysis for the effects of probiotics on FBG

Subgroup	No. of trials	No. of participants	*I*^2^ (%)	*P*_heterogeneity_	Pooled SMD [95% CI]	*P*_effect_	*P*_subgroup_
Race							
Asian	9	722	18.5	0.279	− 0.217 [− 0.364, − 0.070]	0.004	0.020
Caucasian	19	1000	24.8	0.157	− 0.448 [− 0.575, − 0.322]	< 0.001	
Dose of probiotics							
≤ 1 × 10^10^ CFU/day	12	643	38.8	0.082	− 0.335 [− 0.500, − 0.170]	0.003	0.412
> 1 × 10^10^ CFU/day	13	885	0.0	0.203	− 0.248 [− 0.376, − 0.119]	< 0.001	
Duration of intervention							
≤ 8 weeks	16	783	0.0	0.541	− 0.300 [− 0.441, − 0.158]	< 0.001	0.506
> 8 weeks	14	1044	52.9	0.010	− 0.401 [− 0.588, − 0.215]	< 0.001	
Genus of probiotics							
*Lactobacillus*	11	472	0.0	0.669	− 0.194 [− 0.376, − 0.012]	0.037	0.040
*Bifidobacterium*	2	160	69.9	0.068	− 0.626 [− 1.221, − 0.030]	0.039	
*Lactobacillus* and *Bifidobacterium*	15	1071	32.1	0.111	− 0.346 [− 0.498, − 0.195]	< 0.001	
Type of vehicle used to deliver the probiotics							
Powder/capsule/tablet	15	1023	36.4	0.078	− 0.357 [− 0.481, − 0.232]	< 0.001	0.809
Food	13	717	35.1	0.102	− 0.333 [− 0.481, − 0.184]	< 0.001	
Baseline BMI							
< 30 kg/m^2^	16	995	2.8	0.420	− 0.218 [− 0.343, − 0.092]	0.001	0.007
≥ 30 kg/m^2^	12	681	40.6	0.070	− 0.490 [− 0.644, − 0.336]	< 0.001	

FBG, fasting blood glucose; CFU, colony-forming units; BMI,body mass index

#### Effects on insulin

Eight hundred and eighty-six patients in 17 RCTs were included in the meta-analysis of the effects of probiotic intake on insulin level. Probiotic supplementation in T2DM patients led to a significant reduction in insulin level (SMD = − 0.185, 95% CI  − 0.313 to − 0.056, *P*_effect_ = 0.004) without heterogeneity (Fig. 2b, *I*^2^ = 0%, *P*_heterogeneity_ = 0.500). Sensitivity analysis also supported the robustness of the results for insulin level (Additional file 4: Fig. S2b).

As shown in Table 3, the magnitude of the reduction was significantly greater in the subgroup of patients taking food-type probiotics (SMD = − 0.386, 95% CI  − 0.592 to − 0.180, *P*_effect_ < 0.001, *P*_subgroup_ = 0.014) than in the subgroup taking non-food (powder/capsule/tablet) types. In addition, no differences were observed between the subgroups of races, probiotic dose, intervention duration, probiotic genus, or baseline BMI (Table 3, *P*_subgroup_ > 0.050).

**Table 3:** Subgroup analysis for the effects of probiotics on insulin

Subgroup	No. of trials	No. of participants	*I*^2^ (%)	*P*_heterogeneity_	Pooled SMD [95% CI]	*P*_effect_	*P*_subgroup_
Race							
Asian	5	313	0.0	0.777	− 0.145 [− 0.367, 0.078]	0.202	0.764
Caucasian	11	513	23.3	0.222	− 0.187 [− 0.352, − 0.022]	0.027	
Dose of probiotics							
≤ 1 × 10^10^ CFU/day	9	516	27.2	0.202	− 0.169 [− 0.343, 0.005]	0.056	0.851
> 1 × 10^10^ CFU/day	6	323	0.0	0.673	− 0.143 [− 0.362, 0.076]	0.202	
Duration of intervention							
≤ 8 weeks	10	478	0.0	0.693	− 0.291 [− 0.463, − 0.120]	0.001	0.071
> 8 weeks	7	408	1.1	0.416	− 0.052 [− 0.247, 0.143]	0.600	
Genus of probiotics							
*Lactobacillus*	6	356	21.1	0.275	− 0.300 [− 0.510, − 0.090]	0.005	0.183
*Lactobacillus* and* Bifidobacterium*	10	584	0.0	0.661	− 0.119 [− 0.282, 0.044]	0.152	
Type of vehicle used to deliver the probiotics							
Powder/capsule/tablet	10	510	0.0	0.630	− 0.049 [− 0.223, 0.125]	0.581	0.014
Food	6	316	0.0	0.744	− 0.386 [− 0.592, − 0.180]	< 0.001	
Baseline BMI							
< 30 kg/m^2^	8	413	0.0	0.732	− 0.261 [− 0.455, − 0.066]	0.009	0.279
≥ 30 kg/m^2^	8	448	31.2	0.179	− 0.112 [− 0.299, 0.075]	0.239	

CFU, colony-forming units; BMI,body mass index

#### Effects on HbA1c

The effects of probiotic interventions on HbA1c level were evaluated in 23 RCTs including 1,466 T2DM patients. A significant decrease was observed in the HbA1c level in the probiotic group (Fig. 2c, SMD = − 0.421, 95% CI  − 0.583 to − 0.258, *P*_effect_ < 0.001) with moderate heterogeneity (*I*^2^ = 56%, *P*_heterogeneity_ < 0.001). Sensitivity analysis showed that the results for HbA1 level were stable and reliable (Additional file 4: Fig. S2c).

The subgroup analysis was performed for HbA1c according to races (Asian vs. Caucasian), genera of probiotics (*Lactobacillus* vs. *Bifidobacterium* vs. *Lactobacillus* and *Bifidobacterium*), types of vehicle used to deliver the probiotics (food vs. non-food (powder/capsule/tablet)), and baseline BMI (< 30 kg/m^2^ vs. ≥ 30 kg/m^2^). As shown in Table 4, a significantly greater reduction was observed in the HbA1c level in the subgroups of Caucasians (SMD = − 0.465, 95% CI  − 0.672 to − 0.257, *P*_effect_ < 0.001, *P*_subgroup_ = 0.032), *Bifidobacterium* probiotics (SMD = − 0.913, 95% CI  − 1.387 to − 0.438, *P*_effect_ < 0.001, *P*_subgroup_ = 0.001), food-type probiotics (SMD = − 0.524, 95% CI  − 0.800 to − 0.249, *P*_effect_ < 0.001, *P*_subgroup_ = 0.047), and baseline BMI ≥ 30 kg/m^2^ (SMD = − 0.485, 95% CI  − 0.783 to − 0.188, *P*_effect_ = 0.001, *P*_subgroup_ = 0.018). No differences were observed between the subgroups of probiotic dose or intervention duration (*P*_subgroup_ > 0.050).

**Table 4:** Subgroup analysis for the effects of probiotics on HbA1c

Subgroup	No. of trials	No. of participants	*I*^2^ (%)	*P*_heterogeneity_	Pooled SMD [95% CI]	*P*_effect_	*P*_subgroup_
Race							
Asian	9	722	70.2	0.001	− 0.362 [− 0.647, − 0.077]	0.013	0.032
Caucasian	12	639	39.6	0.077	− 0.465 [− 0.672, − 0.257]	< 0.001	
Dose of probiotic							
≤ 1 × 10^10^ CFU/day	8	368	5.0	0.392	− 0.337 [− 0.573, − 0.102]	< 0.001	0.076
> 1 × 10^10^ CFU/day	11	833	62.8	0.003	− 0.484 [− 0.699, − 0.270]	0.005	
Duration of intervention							
≤ 8 weeks	11	543	0.0	0.586	− 0.509 [− 0.681, − 0.337]	< 0.001	0.077
> 8 weeks	12	923	72.4	< 0.001	− 0.359 [− 0.619, − 0.098]	0.007	
Genus of probiotics							
*Lactobacillus*	7	292	54.1	0.042	− 0.250 [− 0.599, 0.098]	0.159	0.001
*Bifidobacterium*	2	160	50.7	0.154	− 0.913 [− 1.387, − 0.438]	< 0.001	
*Lactobacillus* and* Bifidobacterium*	12	890	51.0	0.021	− 0.407 [− 0.605, − 0.209]	< 0.001	
Type of vehicle used to deliver the probiotics							
Powder/capsule/tablet	11	808	55.9	0.012	− 0.384 [− 0.606, − 0.162]	0.001	0.047
Food	10	571	61.5	0.005	− 0.524 [− 0.800, − 0.249]	< 0.001	
Baseline BMI							
< 30 kg/m^2^	13	861	51.1	0.017	− 0.338 [− 0.541, − 0.134]	0.001	0.018
≥ 30 kg/m^2^	8	454	57.6	0.021	− 0.485 [− 0.783, − 0.188]	0.001	

HbA1c, haemoglobin A1c; CFU, colony-forming units; BMI,body mass index

#### Effects on the HOMA-IR score

The results of the meta-analysis of 17 RCTs (*n* = 1,116) suggested significant effects of probiotic interventions on reducing the HOMA-IR scores in T2DM patients (SMD = − 0.224, 95% CI  − 0.342 to − 0.105, *P*_effect_ < 0.001). The heterogeneity (*I*^2^ = 41%, *P*_heterogeneity_ = 0.040) of these RCTs was moderate (Fig. 2b). Sensitivity analysis showed that the pooled effects of probiotic supplementation on HOMA-IR scores did not significantly change, suggesting that the meta-analysis results were stable and reliable (Additional file 4: Fig. S2b).

No statistically significant differences were observed in the HOMA-IR score between subgroups (Table 5, *P*_subgroup_ > 0.050). However, an effective reduction in the HOMA-IR score was observed in the subgroups of Caucasians (SMD = − 0.308, 95% CI  − 0.471 to − 0.146, *P*_effect_ < 0.001, *P*_subgroup_ = 0.173), high baseline BMI (≥ 30 kg/m^2^; SMD = − 0.320, 95% CI  − 0.615 to − 0.026, *P*_effect_ = 0.033, *P*_subgroup_ = 0.144), and *Bifidobacterium* probiotics (SMD = − 0.248, 95% CI  − 0.387 to − 0.109, *P*_effect_ = 0.004, *P*_subgroup_ = 0.345).

**Table 5:** Subgroup analysis for the effects of probiotics on HOMA-IR

Subgroup	No. of trials	No. of participants	*I*^2^ (%)	*P*_heterogeneity_	Pooled SMD [95% CI]	*P*_effect_	*P*_subgroup_
Race							
Asian	5	470	39.0	0.161	− 0.139 [− 0.321, 0.043]	0.134	0.173
Caucasian	11	601	47.3	0.040	− 0.308 [− 0.471, − 0.146]	< 0.001	
Dose of probiotics							
≤ 1 × 10^10^ CFU/day	6	304	69.2	0.006	− 0.179 [− 0.593, 0.234]	0.396	0.969
> 1 × 10^10^ CFU/day	8	653	33.3	0.163	− 0.241 [− 0.438, − 0.043]	0.017	
Duration of intervention							
≤ 8 weeks	8	381	32.7	0.167	− 0.163 [− 0.412, 0.085]	0.198	0.496
> 8 weeks	9	735	52.4	0.032	− 0.312 [− 0.534, − 0.091]	0.006	
Genus of probiotics							
*Lactobacillus*	5	213	43.8	0.130	− 0.101 [− 0.373, 0.170]	0.606	0.345
*Lactobacillus *and* Bifidobacterium*	11	819	48.4	0.036	− 0.248 [− 0.387, − 0.109]	0.004	
Type of vehicle used to deliver the probiotics							
Powder/capsule/tablet	12	882	57.7	0.007	− 0.239 [− 0.453, − 0.026]	0.028	0.912
Food	4	181	0.0	0.829	− 0.230 [− 0.523, 0.063]	0.124	
Baseline BMI							
< 30 kg/m^2^	8	571	11.9	0.338	− 0.165 [− 0.347, 0.017]	0.075	0.144
≥ 30 kg/m^2^	8	466	60.0	0.015	− 0.320 [− 0.615, − 0.026]	0.033	

HOMA-IR, homeostasis model of assessment of insulin resistance, CFU, colony-forming units; BMI,body mass index

### Publication bias analysis

Potential publication bias was assessed using funnel plots and Egger’s test. A visual inspection of the funnel plots revealed no publication bias for FBG, insulin, or HbA1c levels or the HOMA-IR score (Additional file 5: Fig. S3). Egger’s test results showed no publication bias for FBG (*P* = 0.349), insulin (*P* = 0.260) or HbA1c (*P* = 0.108) levels or the HOMA-IR score (*P* = 0.391).

## Discussion

This systematic review and meta-analysis summarised data from 30 RCTs, including a total of 1,827 individuals, to evaluate the effects of probiotic supplementation on glycaemic control in T2DM patients. The results revealed that probiotic supplementation significantly decreased FBG, insulin, and HbA1c levels and HOMA-IR scores in T2DM patients. Further subgroup analyses showed that the effect was larger in the subgroups of Caucasians, high baseline BMI (≥ 30.0 kg/m^2^), *Bifidobacterium* probiotics, and food-type probiotics.

This study supported the notion that probiotics improve glycaemic control in T2DM patients. This is inconsistent with the results reported by the systematic review and meta-analysis of 12 RCTs in 2016 [13]. They found no significant differences in the HbA1c level and HOMA-IR score between the probiotic and control groups of T2DM patients. For their study, the meta-analysis of HbA1c and HOMA-IR were conducted with limited number of RCTs (n = 6), and five of them had the participants’ baseline BMI less than 30 kg/m^2^. In this study, the subgroup analysis found that the effect was larger in individuals with higher baseline BMI (≥ 30.0 kg/m^2^). These may partially explained the differences between the two studies. The gut microbiota is largely involved in the metabolic, nutritional, physiological, and immune functions of the host [49–51]. A previous study showed that T2DM patients are characterised by a decrease in the abundance of certain butyrate-producing bacteria and the enrichment of other microbial functions conferring sulphate reduction and oxidative stress resistance [52]. Changes in the gut microbial composition may be a mechanism whereby probiotic supplementation improves glycaemic control. Probiotic supplementation may modulate and increase the abundance of intestinal flora that are beneficial to glycaemic control [53, 54]. Moreover, the gut microbiota may regulate glucagon-like peptide 1, which promotes the secretion of insulin from islet β cells, and reduces the secretion of glucagon from islet α cells, resulting in a reduction in gastric emptying time, gastrointestinal peristalsis, and loss of appetite [55, 56]. Previous studies have found that probiotics may stimulate the production of short-chain fatty acids, especially butyrate, which increase insulin sensitivity and thus improve glycaemic control [57–59].

The subgroup analyses suggested that *Bifidobacterium* have greater effects than other probiotic genera. Probiotics that colonise the gut may change the host’s gut microbiota. According to a 5-year follow-up study, *Bifidobacterium longum*, a member of the core microbiota of the human gut, can stably colonise the gut [60]. Another study reported that oral supplementation with *B. longum* persists in the gut for 6 months in 30% of subjects [61]. Moreover, Xiao et al. (2020) found that *Bifidobacterium* appears to have a better ability to colonise the gut than *Lactobacillus* [62]. This may explain the finding that *Bifidobacterium* had a larger effect than other probiotic genera on glycaemic-control-related parameters (e.g. FBG and HbA1c levels) in T2DM patients, to some extent, in this study.

Food-type probiotics (e.g. yogurt and fermented milk) may have greater effects than other types of probiotics on glycaemic control in T2DM patients. Gastric acidity is thought to be one of the main obstacles to gut colonisation [63, 64]. Food-type probiotics (e.g. yogurt and fermented milk) may buffer the stomach acid, allowing the probiotics to better colonise the gut [65]. An in vitro study assessed the tolerance of probiotics in the human gastrointestinal tract by evaluating the effects of food addition on the viability of probiotics in simulated pH 2.0 gastric juices, revealing that adding soymilk or a liquid breakfast greatly enhanced the survival of the probiotics [66].

Compared to the baseline BMI < 30 kg/m^2^ subgroup, the stronger beneficial effects of a probiotic intervention were also observed on FBG and HbA1c levels in the baseline BMI ≥ 30.0 kg/m^2^ subgroup. This may be due to gut dysbiosis in obese individuals. In 2021, Liu et al. summarised the characteristics of the gut microbiota in obesity. Obese individuals were observed to have an increased Firmicutes/Bacteroidetes ratio at the phylum level and decreased abundances of the genera *Lactobacillus* and *Bifidobacterium* [67]. Probiotic supplementation may alleviate gut dysbiosis [68]. These findings indicate that obese individuals may be more sensitive to probiotic interventions. In addition, this may partly explain the observed racial differences, i.e. the effect was larger in Caucasians than in Asians. In this study, the average baseline BMI (30.3 kg/m^2^) was higher in Caucasians than in Asians (26.2 kg/m^2^).

In addition, no significant difference was observed between the longer-term intervention (> 8 weeks) and the shorter-term intervention (≤ 8 weeks) groups. In 2020, an RCT was conducted in 150 new-borns (38–40 weeks gestational age). In that study, the intervention group received probiotic supplementation containing 2 × 10^6^ CFU/day of *B. breve* PB04 and *L. rhamnosus* KL53A. The stool samples from days 5, 6, and 30 were collected for an analysis of the gut microbiome. The results showed that *L. rhamnosus* and *B. breve* colonised rapidly, generally on days 5 and 6 [69]. This ability of the probiotics to rapidly colonise the gut may have resulted in the very small difference between the short and long intervention durations.

Furthermore, no significant differences were found between the higher-dose (> 1 × 10^10^ CFU/day) and lower-dose (≤ 1 × 10^10^ CFU/day) probiotic intervention groups. Several studies have reported similar results. Ibarra et al. (2018) performed a randomised double-blind, placebo-controlled trial to determine the effects of 4 weeks of supplementation with 1 × 10^9^ or 1 × 10^10^ CFU of *B. animalis* subsp. *lactis* HN019 on adults diagnosed with functional constipation. The results showed no significant difference between the two groups with different doses of probiotics [70]. However, Whorwell et al. (2006) conducted a multi-centre clinical trial of 362 patients with irritable bowel syndrome (IBS) and found that 1 × 10^8^ CFU of *B. infantis* 35,624 significantly alleviated the symptoms of IBS and that its effect was superior to that of the administration of 1 × 10^6^ CFU/day and 1 × 10^10^ CFU/day of *B. infantis* 35624 [71]. In all of the included RCTs, the probiotic intervention doses were higher than 1 × 10^6^ CFU/day, and only one RCT had a probiotic intervention dose lower than 1 × 10^8^ CFU/day. Thus, these two doses were not used as the limits for subgroup analysis in this systematic review and meta-analysis. Further studies are warranted to determine the optimal dose of probiotics for glycaemic control in T2DM patients.

This study systematically and comprehensively evaluated the effects of probiotic supplementation on glycaemic control in T2DM patients. To the best of our knowledge, this is the first systematic review and meta-analysis study to investigate the differences in the effects of probiotic interventions on glycaemic control in T2DM patients according to participant characteristics (e.g. race, baseline BMI), and intervention characteristics, (e.g. probiotic doses, probiotic genus, treatment duration, and types of vehicles used to deliver the probiotics). However, this study also has some limitations. First, as 12 of the included studies (40%) were conducted in Iran, some racial and ethnic groups may be underrepresented. This may have resulted in a limited racial representation. Second, the number of RCTs in some subgroup analyses was low. For example, in the subgroup analysis of HbA1c level, the number of RCTs in the *Bifidobacterium* subgroup was only two. Third, the duration of most of the RCTs included in the analysis was from 4 to 24 weeks, and only one RCT was longer than 24 weeks (a 36-week intervention). Therefore, the long-term effects could not be explored in this study.

## Conclusions

The findings of this study indicate that probiotic supplementation had favourable effects on glycaemic control in T2DM patients. *Bifidobacterium* and food-type probiotics had greater glucose-lowering effects than other probiotic genera and types of vehicle used to deliver the probiotics. Patients with a higher BMI may gain more glycaemic control benefits from a probiotic intervention. The administration of probiotics may be a promising adjuvant therapy for glycaemic control in T2DM patients.

## Supplementary Information

**Additional file 1: Table S1.** PRISMA Checklist.

**Additional file 2: Table S2.** MeSH and non-MeSH terms used in the systematic search.

**Additional file 3: Figure S1.** (a) Risk of bias summary and (b) risk of bias graph.

**Additional file 4: Figure S2.** Sensitivity analysis for studies included in this meta-analysis. (a) FBG, (b) Insulin, (c) HbAc1, and (d) HOMA-IR.

**Additional file 5: Figure S3.** Funnel plot for studies included in this meta-analysis. (a) FBG, (b) Insulin, (c) HbAc1, and (d) HOMA-IR.

## Data Availability

The data used in this study can be obtained by contacting the corresponding author.

## Abbreviations

T2DM: Type 2 diabetes mellitus
SMD: Standardised mean difference
FBG: Fasting blood glucose
HbA1c: Haemoglobin A1c
HOMA-IR: Homeostasis model of assessment of insulin resistance
RCTs: Randomised controlled trials
BMI: Body mass index
SD: Standard deviation
SE: Standard error
CI: Confidence interval
CFU: Colony-forming units
IBS: Irritable bowel syndrome
