# Supplementary material for: The effects of probiotics supplementation on glycaemic control among adults with type 2 diabetes mellitus: a systematic review and meta-analysis of randomised clinical trials
Source: J Transl Med. 2023 Jul 6;21:442. doi: 10.1186/s12967-023-04306-0 (PMC10324246; doi:10.1186/s12967-023-04306-0)
Supplement: Supplementary file 2 — Additional file 2: Table S2. MeSH and non-MeSH terms used in the systematic search. [file 12967_2023_4306_MOESM2_ESM.doc]

Supplementary table S2 MeSH and non-MeSH terms used in the systematic search

| **Database** |  | **Syntax** | **Results** |
| --- | --- | --- | --- |
| PubMed |  | (((humans) AND ((((((((((Randomized Controlled Trial) OR (random*)) OR (control*)) OR (Placeboes)) OR (Placebos)) OR (clinical trials)) OR (Clinical)) OR (Trials)) OR (Trialed)) OR (Trialing))) AND ((((((((((Probiotics) OR (Probiotic)) OR (Lactobacillus)) OR (Bifidobacterium)) OR (Saccharomyces)) OR (Streptococcus)) OR (Enterococcus)) OR (Propionibacterium)) OR (Kefir)) OR (Yogurt))) AND (((((((((((((diabetes mellitus, type 2) OR (diabetes mellitus type 2 diabetes)) OR (mellitus noninsulin dependent)) OR (Ketosis-Resistant Diabetes Mellitus)) OR (diabetes mellitus non insulin dependent)) OR (Non-Insulin-Dependent Diabetes Mellitus)) OR (Stable Diabetes Mellitus)) OR (diabetes mellitus type II)) OR (Type 2 Diabetes Mellitus)) OR (Type 2 Diabetes)) OR (diabetes type 2)) OR (T2DM)) OR  (T2D)) | 657 |
| Web Of Sciences  （BIOSIS, CSCD, D IIDW, INSPEC, KJD  , MEDLINE, SCIEL O, WOS） |  | #4 AND #3 AND #2 AND #1 | 1234 |
|  | #1 | ((((((((((((TS=(diabetes mellitus, type 2 )) OR TS=(diabetes mellitus type 2 diabetes )) OR TS=(mellitus noninsulin dependent)) OR TS=(Ketosis-Resistant Diabetes Mellitus)) OR TS=(diabetes mellitus non insulin dependent)) OR TS=(Non-Insulin-Dependent Diabetes Mellitus)) OR TS=(Stable Diabetes Mellitus)) OR TS=(diabetes mellitus type Ⅱ)) OR TS=(Type 2 Diabetes Mellitus)) OR TS=(Type 2  Diabetes)) OR TS=(diabetes type 2)) OR TS=(T2DM)) OR TS=(T2D) | [468,087](https://www.webofscience.com/wos/alldb/summary/47d43216-e22b-41af-b93e-a3cb8ee02ded-55c86ad5/relevance/1) |
|  | #2 | (((((((((TS=(Probiotics)) OR TS=(Probiotic)) OR TS=(Lactobacillus)) OR TS=(Bifidobacterium)) OR TS=(Saccharomyces)) OR  TS=(Streptococcus)) OR TS=(Enterococcus)) OR TS=(Propionibacterium)) OR TS=(Kefir)) OR TS=(Yogurt) | [748,151](https://www.webofscience.com/wos/alldb/summary/8c28b55d-9c50-48ee-8eac-9aa2258972eb-55c88fa6/relevance/1) |
|  | #3 | human | [29,682,356](https://www.webofscience.com/wos/alldb/summary/5df08b9d-a106-4cd7-8747-a8f0a650dd8c-55e60916/relevance/1) |
|  | #4 | ((((TS=(Randomized Controlled Trial)) OR TS=(Random*)) OR TS=(Control*)) OR TS=(Placebo)) OR TS=(trial) | [28,639,974](https://www.webofscience.com/wos/alldb/summary/c0171cff-7412-4f4a-9f22-0e5ad3dcbc4f-55e6425e/relevance/1) |
| Embase |  | #1 AND #11 AND #12 AND #13 | 1,376 |
|  | #1 | 'non insulin dependent diabetes mellitus'/exp OR 'non insulin dependent diabetes mellitus' OR 'adult onset diabetes'/exp OR 'adult onset diabetes' OR 'adult onset diabetes mellitus'/exp OR 'adult onset diabetes mellitus' OR 'diabetes mellitus type 2'/exp OR 'diabetes mellitus type 2' OR 'diabetes mellitus type ii'/exp OR 'diabetes mellitus type ii' OR 'diabetes mellitus, non-insulin-dependent'/exp OR 'diabetes mellitus, non-insulin-dependent' OR 'diabetes mellitus, type 2'/exp OR 'diabetes mellitus, type 2' OR 'diabetes mellitus, type ii'/exp OR 'diabetes mellitus, type ii' OR 'diabetes mellitus, maturity onset'/exp OR 'diabetes mellitus, maturity onset' OR 'diabetes mellitus, non insulin dependent'/exp OR 'diabetes mellitus, non insulin dependent' OR 'diabetes type 2'/exp OR 'diabetes type 2' OR 'diabetes type ii'/exp OR 'diabetes type ii' OR 'diabetes, adult onset'/exp OR 'diabetes, adult onset' OR 'dm 2'/exp OR 'dm 2' OR 'insulin independent diabetes'/exp OR 'insulin independent diabetes' OR 'insulin independent diabetes mellitus'/exp OR 'insulin independent diabetes mellitus' OR 'ketosis resistant diabetes mellitus'/exp OR 'ketosis resistant diabetes mellitus' OR 'maturity onset diabetes'/exp OR 'maturity onset diabetes' OR 'maturity onset diabetes mellitus'/exp OR 'maturity onset diabetes mellitus' OR 'maturity onset diabetes of the young'/exp OR 'maturity onset diabetes of the young' OR 'niddm'/exp OR 'niddm' OR 'non insulin dependent diabetes'/exp OR 'non insulin dependent diabetes' OR 'noninsulin dependent diabetes'/exp OR 'noninsulin dependent diabetes' OR 'noninsulin dependent diabetes mellitus'/exp OR 'noninsulin dependent diabetes mellitus' OR 'type 2 diabetes'/exp OR 'type 2 diabetes' OR 'type 2 diabetes mellitus'/exp OR 'type 2  diabetes mellitus' OR 'type ii diabetes'/exp OR 'type ii diabetes' | 367416 |
|  | #2 | 'probiotic agent'/exp OR 'probiotic agent' OR 'probiotic'/exp OR probiotic OR 'probiotics'/exp OR probiotics | [57,797](https://www.embase.com/) |
|  | #3 | 'lactobacillus'/exp OR lactobacil* OR betabacterium OR lactobacileae OR lactobacilleae OR lactobacilli OR lactobacteria | [70,347](https://www.embase.com/) |
|  | #4 | 'bifidobacterium'/exp OR bifidobacteri* | [24,384](https://www.embase.com/) |
|  | #5 | 'saccharomyces'/exp OR saccharomyc* | [129,515](https://www.embase.com/) |
|  | #6 | 'streptococcus'/exp OR streptococc* | [203,190](https://www.embase.com/) |
|  | #7 | 'enterococcus'/exp OR enterococc* | [72,128](https://www.embase.com/) |
|  | #8 | 'propionibacterium'/exp OR propionibacteri* OR 'bacterium acidi propionici' | [14,754](https://www.embase.com/) |
|  | #9 | 'kefir'/exp OR kefir OR kephir | [1,193](https://www.embase.com/) |
|  | #10 | 'yoghurt'/exp OR yoghurt OR yoghourt OR yogurt OR zabadi | [8,349](https://www.embase.com/) |

|  | #11 | 'randomized controlled trial'/exp OR random* OR control* OR trial | [14,411,019](https://www.embase.com/) |
| --- | --- | --- | --- |
|  | #12 | 'human'/de | [25,196,125](https://www.embase.com/) |
|  | #13 | #2 OR #3 OR #4 OR #5 OR #6 OR #7 OR #8 OR #9 OR #10 | 489,487 |
| Cochrane Library | #1 | diabetes mellitus, type 2 | 63044 |
|  | #2 | diabetes mellitus type 2 diabetes | 63044 |
|  | #3 | mellitus noninsulin dependent | 692 |
|  | #4 | Ketosis-Resistant Diabetes Mellitus | 2 |
|  | #5 | diabetes mellitus non insulin dependent | 20117 |
|  | #6 | Non-Insulin-Dependent Diabetes Mellitus | 19271 |
|  | #7 | Stable Diabetes Mellitus | 4077 |
|  | #8 | diabetes mellitus type Ⅱ | 6925 |
|  | #9 | Type 2 Diabetes Mellitus | 63044 |
|  | #10 | Type 2 Diabetes | 82591 |
|  | #11 | diabetes type 2 | 82591 |
|  | #12 | T2DM | 7559 |
|  | #13 | T2D | 4083 |
|  | #14 | #1 OR #2 OR #3 OR #4 OR #5 OR #6 OR #7 OR #8 OR #9 OR #10 OR #11 OR #12 OR #13 | 84877 |
|  | #15 | Probiotics | 6669 |
|  | #16 | Probiotic | 6386 |
|  | #17 | Lactobacillus | 5920 |
|  | #18 | Bifidobacterium | 3263 |
|  | #19 | Saccharomyces | 648 |
|  | #20 | Streptococcus | 5458 |
|  | #21 | Enterococcus | 1258 |
|  | #22 | Propionibacterium | 465 |
|  | #23 | Kefir | 86 |
|  | #24 | Yogurt | 1587 |
|  | #25 | #15 OR #16 OR #17 OR #18 OR #19 OR #20 OR #21 OR #22 OR #23 OR #24 | 18014 |
|  | #26 | #14 AND #25 in Trials | 781 |
