# Supplementary material for: The effects of probiotics supplementation on glycaemic control among adults with type 2 diabetes mellitus: a systematic review and meta-analysis of randomised clinical trials
Source: J Transl Med. 2023 Jul 6;21:442. doi: 10.1186/s12967-023-04306-0 (PMC10324246; doi:10.1186/s12967-023-04306-0)
Supplement: Supplementary file 3 — Additional file 3: Figure S1. (a) Risk of bias summary and (b) risk of bias graph. [file 12967_2023_4306_MOESM3_ESM.pdf]

a

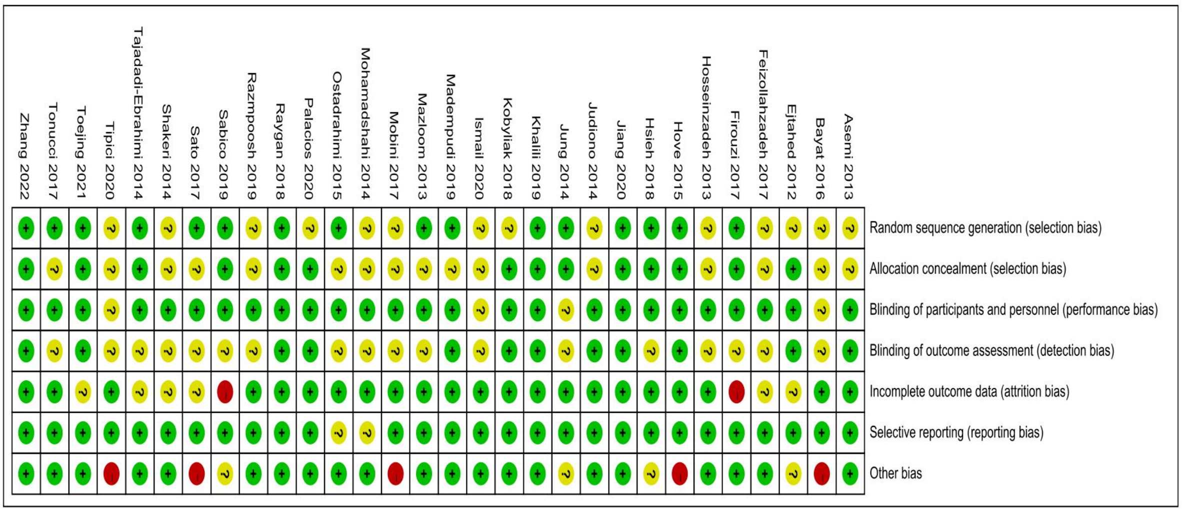

b

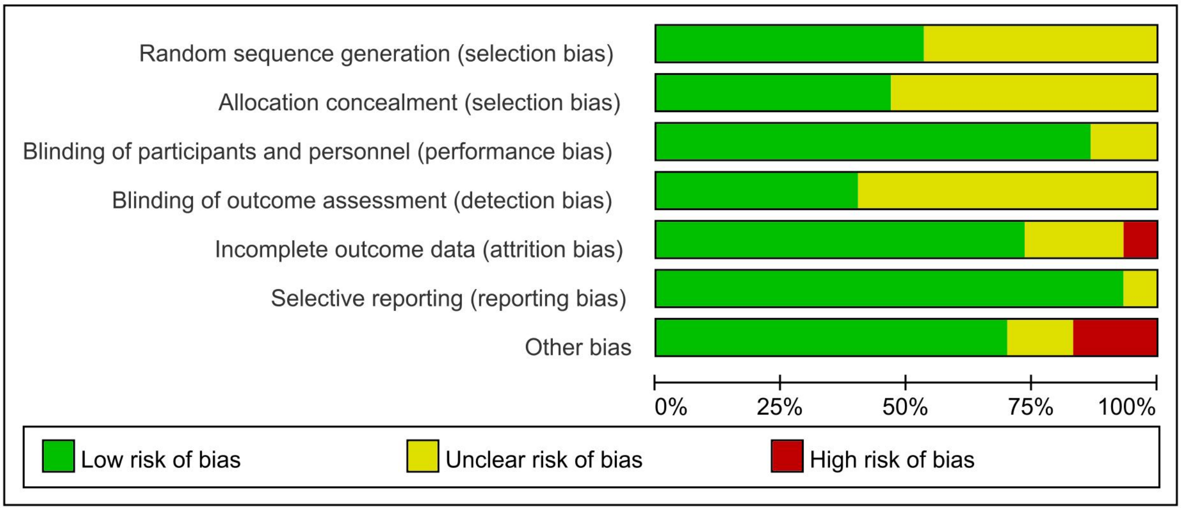

Supplementary figure S1 (a) risk of bias summary and (b) risk of bias graph
