# Supplementary material for: The effects of probiotics supplementation on glycaemic control among adults with type 2 diabetes mellitus: a systematic review and meta-analysis of randomised clinical trials
Source: J Transl Med. 2023 Jul 6;21:442. doi: 10.1186/s12967-023-04306-0 (PMC10324246; doi:10.1186/s12967-023-04306-0)
Supplement: Supplementary file 5 — Additional file 5: Figure S3. Funnel plot for studies included in this meta-analysis. (a) FBG, (b) Insulin, (c) HbAc1, and (d) HOMA-IR. [file 12967_2023_4306_MOESM5_ESM.pdf]

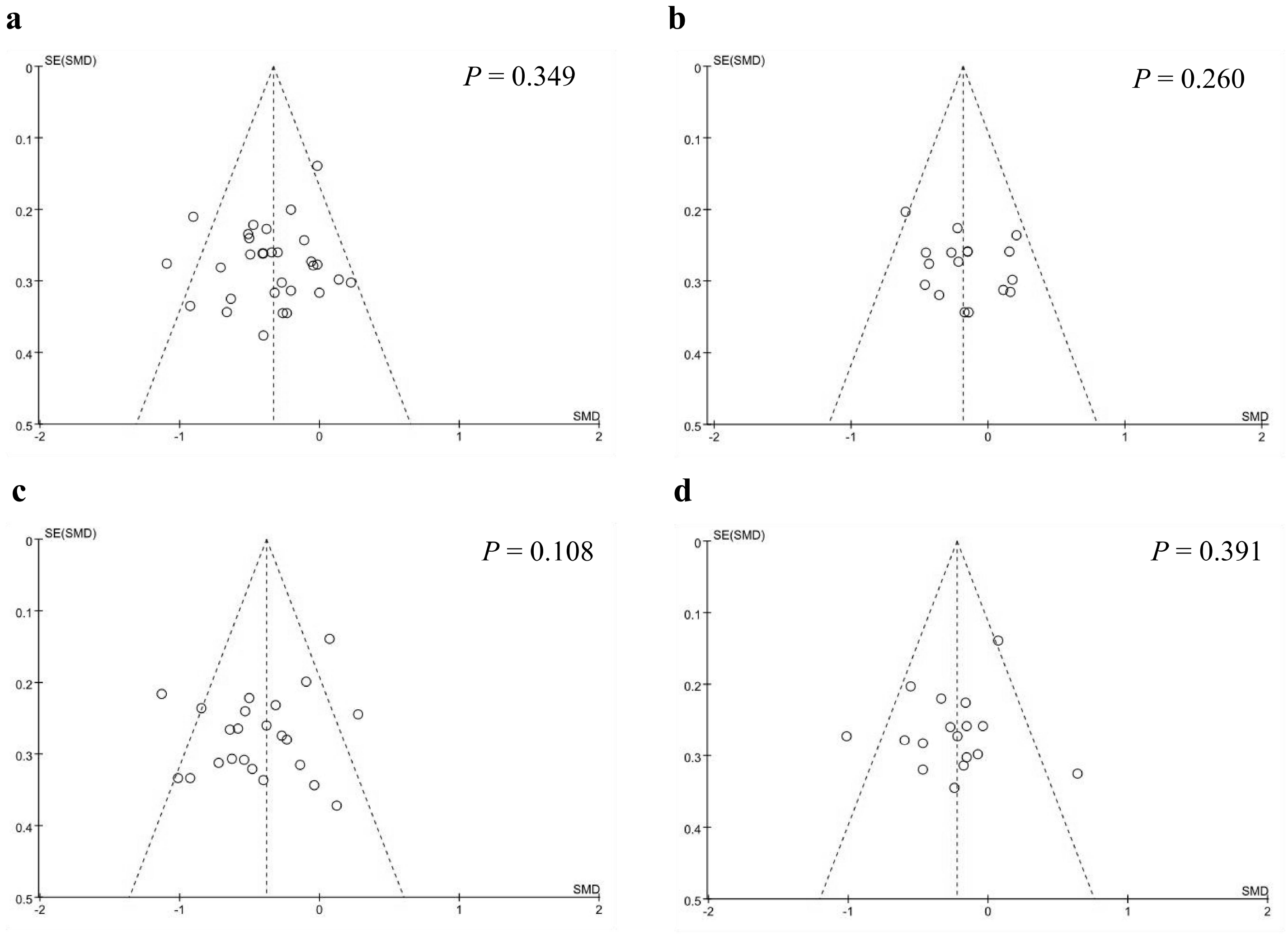

Supplementary figure S3 Funnel plot for studies included in this meta-analysis. (a) FBG, (b) Insulin, (c) HbAc1, and (d) HOMA-IR
